# A Nanoscale Structure Based on an MIM Waveguide Coupled with a Q Resonator for Monitoring Trace Element Concentration in the Human Body

**DOI:** 10.3390/mi12111384

**Published:** 2021-11-11

**Authors:** Tingsong Li, Shubin Yan, Pengwei Liu, Xiaoyu Zhang, Yi Zhang, Lifang Shen, Yifeng Ren, Ertian Hua

**Affiliations:** 1 School of Electrical and Control Engineering, North University of China, Taiyuan 030051, China; lts15296737639@163.com (T.L.); lpw18834800530@163.com (P.L.); zhangxiaoyu9725@163.com (X.Z.); Renyifeng126@126.com (Y.R.); 2 School of Electrical Engineering, Zhejiang University of Water Resources and Electric Power, Hangzhou 310018, China; zhangyi@zjweu.edu.cn (Y.Z.); shenlf@zjweu.edu.cn (L.S.); het@zjweu.edu.cn (E.H.); 3 Joint Laboratory of Intelligent Equipment and System for Water Conservancy and Hydropower Safety Monitoring of Zhejiang Province and Belarus, Hangzhou 310018, China

**Keywords:** MIM, surface plasmon polaritons (SPPs), Fano resonance, refractive index sensor, trace element concentration monitoring

## Abstract

In this study, a nano-refractive index sensor is designed that consists of a metal–insulator–metal (MIM) waveguide with a stub-1 and an orthogon ring resonator (ORR) with a stub-2. The finite element method (FEM) was used to analyze the transmission characteristics of the system. We studied the cause and internal mechanism of Fano resonance, and optimized the transmission characteristics by changing various parameters of the structure. In our experimental data, the suitable sensitivity could reach 2260 nm/RIU with a figure of merit of 211.42. Furthermore, we studied the detection of the concentration of trace elements (such as Na^+^) of the structure in the human body, and its sensitivity reached 0.505 nm/mgdL^−1^. The structure may have other potential applications in sensors.

## 1. Introduction

Common optical refractive index sensors include optical fiber sensors [1,2], photonic crystal sensors, and surface plasmon resonance (SPR) sensors [3,4]. Although the development of optical refractive index sensors is relatively mature, due to the limitation of the optical diffraction limit, the size of an optical refractive index sensor has a certain limit. How to break through the optical diffraction limit, realize the enhancement of electromagnetic energy at subwavelength size, and further realize the miniaturization and integration of optical devices has become a popular research problem. For this reason, surface plasmon polaritons (SPPs) have begun to attract the attention of scientists.

SPPs are a form of collective oscillation in which incident light interacts with free electrons on a metal surface and can transmit along the interface [5,6]. It can break through the diffraction limit, so as to realize the regulation and transmission of light at subwavelength size [7,8]. Various optical devices based on SPPs have also been created [9], including filters [10,11], refractive index sensors [12,13,14,15,16], optical switches [17,18], and slow light devices [19]. Fano resonance also occurs in the coupling progress of SPPs. Fano resonance results from the coupling of the wider continuous state with the narrower discrete state. Unlike Lorentz resonance, Fano resonance is typically sharp and asymmetrically linear. Due to such characteristics, it has a high space electromagnetic field constraint ability and can better distinguish small frequency shifts, which gives the surrounding environment sensitivity better performance. In order to obtain a better Fano resonance curve to reach a higher refractive index (i.e., sensitivity) and figure of merit (FOM) values, a variety of MIM waveguide-based structures continue to appear, such as in Yi et al., who presented a tunable Fano resonance system with a CSRR structure [20]. Zhang et al. designed an MIM waveguide with ring splitting cavity, whose sensitivity and FOM were 1200 nm/RIU and 122, respectively [21]. Ren et al. designed a plasma refractive index sensor, which can realize the double Fano resonance and adjust the curve by changing the parameters [22]. All of this literature has a common feature, that is, inserting a clearance or introducing a baffle to obtain an asymmetric structure, which can improve the performance of the system.

Basic on this idea, a simple nano refractive index sensor is here designed, which is constituted by a bus waveguide with a stub-1 and an orthogon ring resonator (ORR) with a stub-2. To be noticed is that an orthogon has a longer perimeter than a circle for the same area. This means that the orthogon ring resonator has a higher coupling efficiency than the circular resonator because of the longer coupling distance. Its propagation characteristics are demonstrated by the finite element method (FEM). The causes of Fano resonance under an asymmetric structure (ORRS) and the influence of different structural parameters on the system’s performance are studied here.

## 2. Materials and Methods

**Figure 1:**
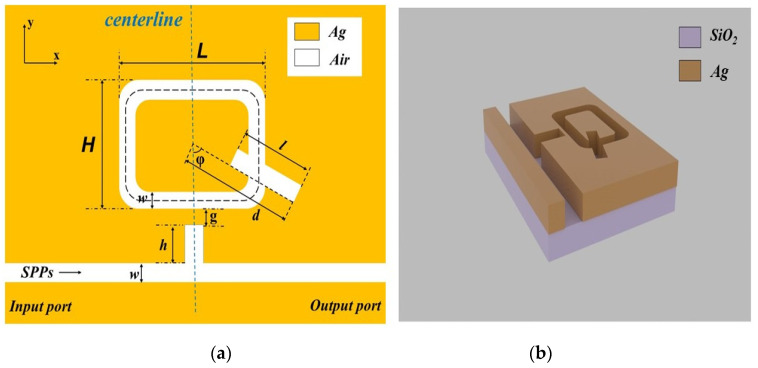
(**a**) 2D plan diagram of the structure; (**b**) 3D stereogram of the structure.

We used COMSOL Multiphysics to establish the structure model. The FEM is used to analyze the transmission characteristics of the SPPs. At the top and bottom of the structure, we set the boundary conditions of the perfectly matched layer to absorb the overflow wave, along with a superfine mesh division to ensure the perfect subdivision of the structure and better simulation accuracy. The z-dimension of the metal is much larger than the light wavelength. Hence, we could build a 2D model to approximate the 3D model. The two-dimensional structure is shown in Figure 1a, and the three-dimensional model structure is shown in Figure 1b. The 2D structure is made up of a bus waveguide with a stub-1 and an orthogon ring resonator (ORR) with a stub-2. For convenience, ORR with a stub-2 is called ORRS. The length and width of ORR are *L* and *H*. The length of stub-2 on ORR is *l.* The distance between the base angle of stub-2 and the center of ORR is *d*, and the included angle is *φ*. The distance between the ORR and stub-1 is *g*. The height of stub-1 is *h*. In order for the system to only excite the fundamental transverse magnetic (TM_0_) mode, we left the width parameters of the ORRS and bus waveguide unchanged (i.e., *w* = 50 nm) [23]. Its dispersion relation is defined as [24]:

(1) $$\tanh\left(k\omega \right)=-\frac{2k\alpha_{c}}{k^{2}+p^{2}\alpha_{c}}$$

where k and $k_{0}=2\pi /\lambda_{0}$ represent the wave vector in the waveguide and the wave vector in free space, respectively. Furthermore, $p=\varepsilon_{\mathrm{in}}/\varepsilon_{m}$, $\alpha_{c}={\left[k_{0}^{2}\left(\varepsilon_{in}-\varepsilon_{m}\right)+k\right]}^{\frac{1}{2}}$. $\varepsilon_{in}$ and $\varepsilon_{m}$ are the permittivity of the medium and the metal, respectively.

The white and orange areas represent air and silver, respectively. Silver was chosen as a filler metal because of its low power consumption—namely, the electromagnetic response of silver is small relative to the imaginary part of the dielectric constant, ensuring a strong magnetic field in the waveguide and resonator. The MIM waveguide structure is easy to fabricate; it is produced by depositing 100 nm silver on a SiO_2_ substrate by chemical vapor deposition. Other structures can be etched onto the silver layer by an electron beam, and the remaining silver layer can be removed by chemical etching with dilute nitric acid and water. The relative dielectric constant of silver is defined by the Debye–Drude dispersion model [25]:

(2) $$\varepsilon \left(\omega \right)=\varepsilon_{\infty }+\frac{\varepsilon_{s}-\varepsilon_{\infty }}{1+i\tau \omega }+\frac{\sigma }{i\omega \varepsilon_{0}}$$

In Formula (2), $\varepsilon_{\infty }=3.8344$ is the relative permittivity of infinite frequencies, $\varepsilon_{s}=-9530.5$ stands for the static permittivity, and the relaxation time (τ) and the conductivity (σ) of silver are $7.35\times 10^{-15}s$ and $1.1486\times 10^{7}\;S/m$, respectively.

Sensitivity *S* is an important performance index to evaluate for the nano-scale refractive index sensor, which reflects the ratio of the refractive index change to the drift of the formant. Its expression is [26]:

(3) S=Δλ/Δn

where Δλ and Δn represent the variation of resonance wavelength and refractive index, respectively.

The figure of merit (*FOM*) value is also an important parameter to characterize the nano-level refractive index sensor. Unlike the sensitivity expressed in the terms of spectral shift, it is based on the intensity variation and can be defined as [27]:

(4) $$FOM=\frac{∆T}{T\times ∆n}$$

In Formula (4), *T* denotes the transmittance and ∆T/∆n denotes the shift in transmission at the specified wavelength induced by ∆n.

## 3. Results

**Figure 2:**
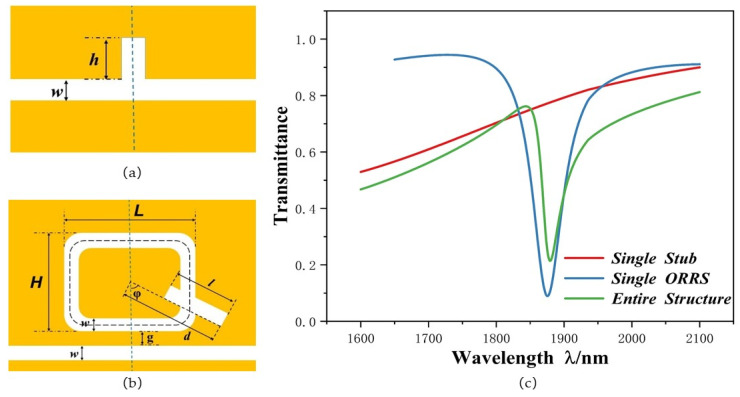
(**a**,**b**) 2D structure plan diagram of single stub and single ORRS; (**c**) Transmittance spectra of the single stub-1 structure (blue line), single ORRS structure (red line), and entire structure (green line).

In order to understand the differences between a single stub-1 structure, a single ORRS structure, and the entire structure, we have drawn their schematic diagrams and transmission spectra. The single stub-2 structure is composed of a stub-2 and bus waveguide, and its schematic diagram is shown in Figure 2a. The single ORRS structure is made up of a bus waveguide and ORRS, and its schematic diagram is depicted in Figure 2b. A schematic diagram of the overall structure is shown in Figure 1b. Their transmittance spectra are depicted in Figure 2c. They have the same structure parameter setting, which is as follows: *L* = 500 nm, *H* = 350 nm, *l* = 250 nm, *d* = 353 nm, *φ* = 60°, *g* = 10 nm, *h* = 140 nm.

The red line in Figure 2c is a continuous curve with high transmittance, so it can be used as a continuous state to generate Fano resonance. The blue line has a transmittance lower than 0.1. Therefore, the dips are deep enough to consider it as a discrete state. The green line is the asymmetric curve of the two structures coupled together, indicating the Fano resonance. It is proved that Fano resonance is produced by coupling a wider continuous state with a narrower discrete state.

**Figure 3:**
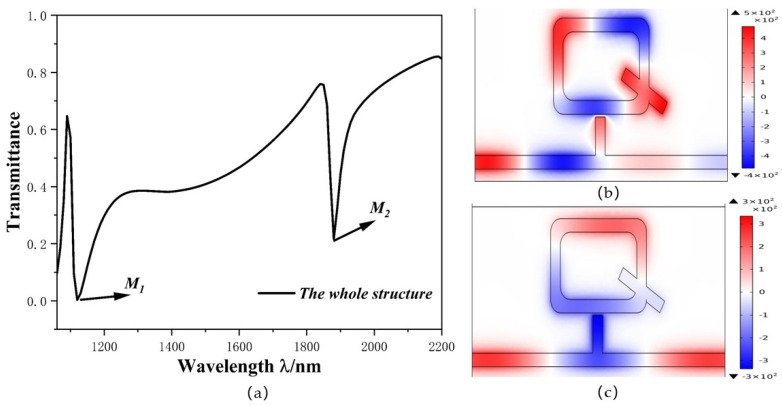
(**a**) Transmission spectra; (**b**,**c**) Diagram of the normalized magnetic field distribution at *λ* = 1110 nm and *λ* = 1880 nm, respectively.

In order to better understand the internal principle of Fano resonance, the transmission spectra of the system and the distributions of the normalized magnetic field $H_{z}$ are depicted in Figure 3a–c. Figure 3b shows the magnetic field distribution of $M_{1}$ (*λ* = 1110 nm). The SPP’s energy is mostly in the left half of the ORRS and MIM waveguide, because there is an antiphase relationship between the upper part of stub-1 and the lower part of ORRS, which leads to destructive interference between the SPPs entering the ORRS and those leaving the ORRS, which inhibits the transmittance. Thus, the transmittance at $M_{1}$ in Figure 3a is very low. Figure 3c shows the magnetic field distribution of $M_{2}$ (*λ* = 1880 nm). Because of the in-phase relationship between the stub-1 structure and ORRS structure, coherent enhancement will occur between them. Consequently, transmittance at $M_{2}$ is slightly higher. Although the transmittance at $M_{2}$ is slightly high than $M_{1}$, the sensitivity of $M_{2}$ reaches 2260 nm/RIU, which is higher than 1200 nm/RIU of $M_{1}.$ Hence, we chose $M_{2}$ as the object of this study.

**Figure 4:**
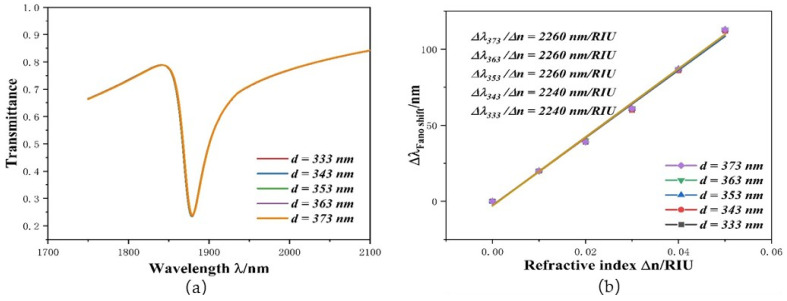

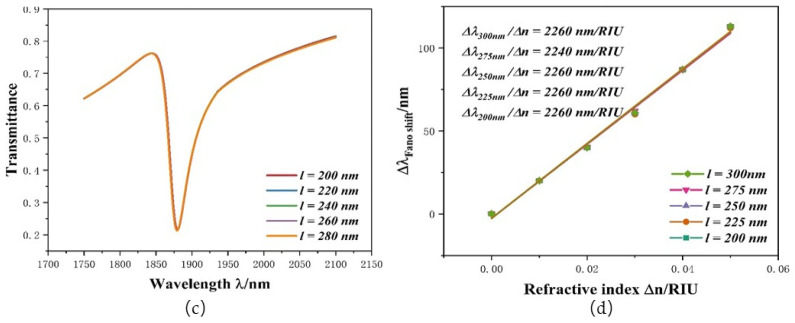
(**a**) Transmission spectra at disparate *d* values; (**b**) *S* changes under diverse *d* values; (**c**) Transmission spectra at disparate *l* values; (**d**) *S* changes under diverse *l* values.

In the ORRS structure, the first thing to study is whether the position and the size of stub-2 affect *S* and the *FOM* value. First, the position of stub-2 can be achieved by changing the size of the *d* parameter. The stub-2 structure with different *d*s, set to 333 nm, 343 nm, 353 nm, 363 nm, and 373 nm, was studied, while other parameter settings were the same as in Figure 2 (the values of parameters not mentioned in the following studies are the same as these). Its transmittance spectra and *S*-changing image are depicted in Figure 4a,b. Subsequently, the same operation was performed, but the value of *l* was increased from 200 nm to 300 nm, at 25 nm intervals, and a total of five sets of data were produced, to study the effect of stub-2 size on performance. The results are shown in Figure 4c,d. As plotted in Figure 4a, despite the difference of *d*, the images are coincident. This proves that different *d* values have the same transmittance and wavelength: the transmission is as low as 0.234 and *λ* = 1879 nm. As shown in Figure 4b, although the *d* value was changing, the sensitivity fitting curves basically coincide. There could have been a deviation of 1 nm at some points, resulting in a sensitivity change of 20 nm/RIU. These deviations have a negligible effect on performance. According to Formula (4), when *T* and ∆T/∆n are the same, the *FOM* value is also the same. The result of the analysis of *l* is the same as that of *d*: its transmission is 0.216 nm, *λ* = 1880 nm, and different *l* values have the same *S* and *FOM*. Based on such results, we can exclude the interference of *d* and *l* when studying the influence of other parameters on system performance.

**Figure 5:**
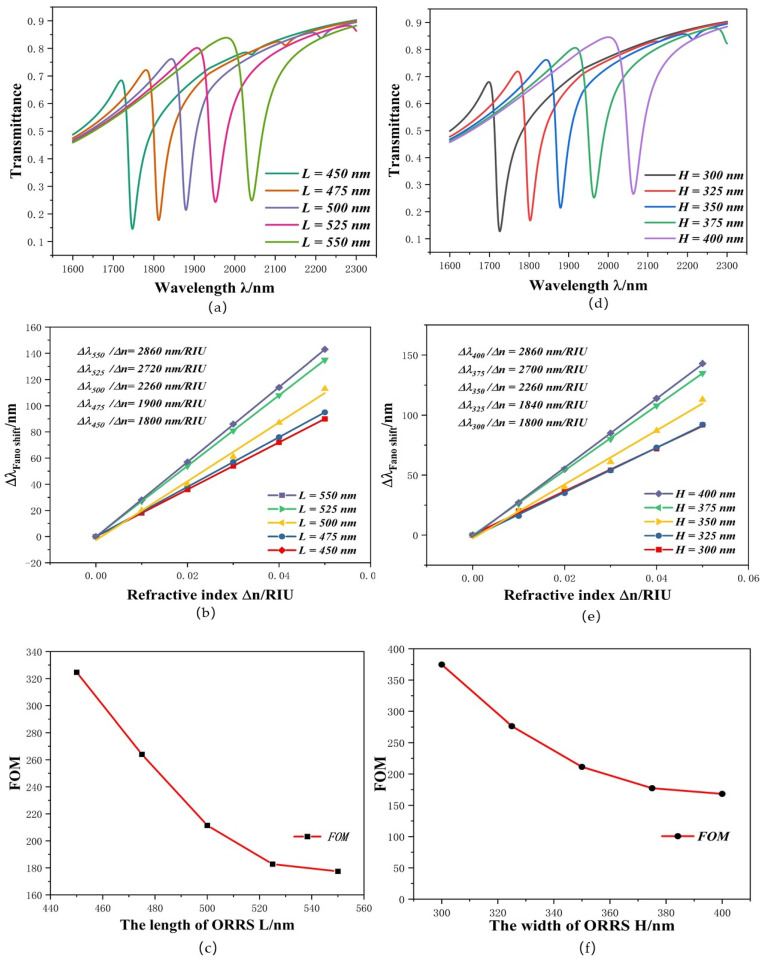
(**a**) ORRS transmission spectra under different *L* values; (**b**) *S* changes under different *L* values; (**c**) *FOM* changes under different *L* values; (**d**) SPPS transmission spectra under different *H* values; (**e**) *S* changes under different *H* values; (**f**) *FOM* changes under different *H* values.

Subsequently, we studied the effect of geometric parameters *L* and *H* on system performance. The different *L* parameters of ORRS were set as follows: 450 nm, 475 nm, 500 nm, 525 nm, and 550 nm, and its transmission spectra and sensitivity changes are shown in Figure 5a,b. With the gradual increase in *L*, the curve presents an obvious redshift, the position of the dip moved from 1747 nm to 2042 nm and S increased from 1800 nm/RIU to 2860 nm/RIU As shown in Figure 5c, as *L* increases, the change in *FOM* decreases from 324.65 to 177.41. The maximum value of FOM = 324.65 is achieved at *L* = 450 nm, but its sensitivity is only up to 1800 nm/RIU. We then enacted the same setting with the values of *H* at 300 nm, 325 nm, 350 nm, 375 nm, and 400 nm. The results are shown in Figure 5d–f. The change in *H* has the same influence on system performance as *L*. The curve also appears to redshift; when the *H* of the ORRS increases, the position of the dip moved from 1725 nm to 2065 nm and the transmittance (*T*) increased from 0.217 to 0.265. According to the *FOM* formula, a change in *T* value will cause a change in *FOM* value. This change can be observed in Figure 5f.

The curve redshifted as *L* and *H* increased, on the one hand, because ORRS plays a role in the structure as a narrow discrete state that generates Fano resonance; when its structure changes, it affects the Fano curve to some extent. On the other hand, we can also explain this phenomenon using standing wave theory (λm=2Re(neff)L1/(m−∅/m), where *L*_1_ is the effective length. SPPs enter the ORRS through the MIM waveguide and stub-1. Assuming that SPPs are coupled clockwise along the ORRS and then returns to the MIM waveguide along the original route, the effective length can be expressed as: $L_{1}$ = 2 (*L* + *H*). When the effective length increases, the resonant wavelength also increases, resulting in the phenomenon of curve redshift. Therefore, we need to consider various factors and choose appropriate structural parameters. Thus, the most appropriate values for *L* and *H* are *L* = 500 nm and *H* = 350 nm, and the maximum *FOM* is 211.42. The *S* value at this point is 2260 nm/RIU. Its performance was better than most of the parameters in Table 1 [28,29,30,31].

**Figure 6:**
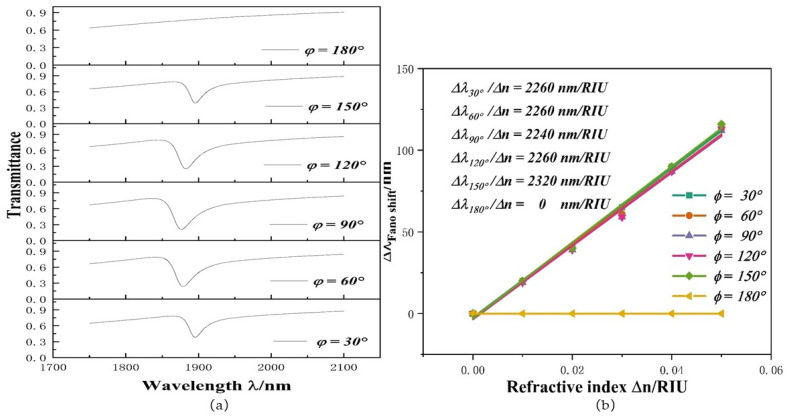
(**a**) Transmission spectra at various angles; (**b**) *S* changes under different angles.

If the angle changes and the structure changes, will there be different propagation characteristics? Based on this query, we set the angle values from 30° to 180° at 30° intervals. Its transmission spectrum and *S* changes are shown in Figure 6a,b. The 30° structure and 150° structure have similar transmission spectra. The 60° structure and 120° structure have similar transmission spectra. The dip in the transmission spectra disappears at 180°, because when the stub-2 is at 180°, the symmetry of the whole system’s structure about the centerline leads to a change in transmission characteristics. When the dip disappears, the *S* shows a horizontal straight line (i.e., the sensitivity is 0). If the 180° structure is not considered, the sensitivity values at other angles are close. Although its sensitivity is highest at 150°, transmittance is 0.38. Hence, after comprehensive consideration of all factors, we chose a structure at 60°, whose transmittance is 0.214. To explore whether the system performance changes due to symmetry, the data of ORRS (500 × 350), ORRS without stub-2 and square resonators without stub-2 (420 × 420) are compared in Table 2. Although the orthogon area is slightly larger than the orthogon area, the sensitivity of ORRS without stub-2 is better than that of the square without stub-2. The *S* of ORRS without stub-2 reaches 2200 nm/RIU with a *FOM* of 219.51, but it requires a higher wavelength range (i.e., higher performance lasers). The structure of ORRS achieves better sensitivity and a smaller wavelength range. Therefore, a better performance is achieved by introducing a baffle.

**Figure 7:**
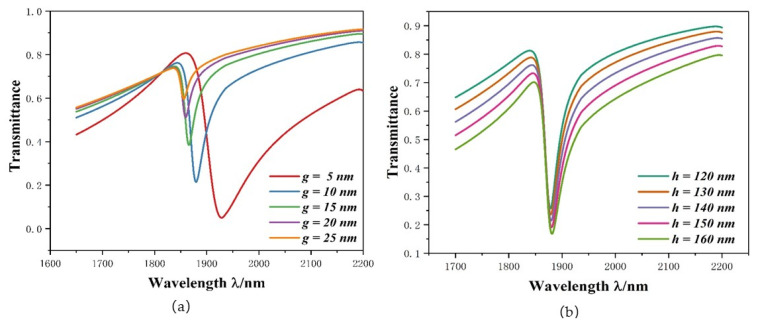
(**a**) Transmission spectra at disparate coupling distances; (**b**) Transmission spectra for different height of stub-1.

Moreover, we studied the influence of the *g* parameter on system performance, setting it as follows: *g* = 5 nm, 10 nm, 15 nm, 20 nm, and 25 nm. As can be observed from Figure 7a, the curve is blueshifted, and its transmittance increased significantly. The reason for this is that as *g* increases, the coupling strength of ORRS and stub-1 weakens, resulting in an increase in transmittance at the dip. This alone does not mean that the minimum *g* value should be selected to ensure transmittance. When *g* = 5 nm, the transmittance is as low as 0.05, but the curve is too large.

Subsequently, the impact of different heights of stub-1 was also investigated. The *h* was changed from 120 nm to 160 nm at intervals of 10 nm, and its transmission spectra are shown in Figure 7b. With the increase in *h*, the position of the dip hardly changes but the transmittance decreases from 0.2573 to 0.1682. When the transmittance value is 0.1682, the sensitivity value is 2180 nm/RIU. Therefore, the appropriate *FOM* and *S* value can be selected by changing *h*.

## 4. Discussion

**Figure 8:**
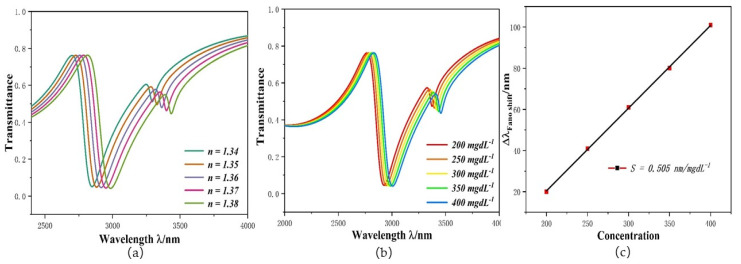
(**a**) Transmission spectra under diverse refractive indexes; (**b**) Transmission spectra under different concentra-tion; (**c**) Sensitivity variation diagram for different concentrations.

The structure designed here can be applied to biosensing. Its principle is that there is a relationship between the concentration of the measured object and the refractive index, and the concentration of the substance can be calculated by the change in the refractive index. Therefore, the variation of system performance according to the refractive index is the first topic to be explored. The refractive index of a biological sample ranges from 1.33 to 1.40. If the structure is to be used in biological sample, its refractive index range should also be limited. Hence, the parameter settings of this structure are as follows: *L* = 500 nm, *H* = 350 nm, *l* = 250 nm, *d* = 353 nm, *φ* = 60°, *g* = 10 nm, *h* = 140 nm, and the *n* is changed from 1.34 to 1.38 at intervals of 0.01. Its transmission spectra are shown in Figure 8a. The curve has an obvious redshift with the increase in the refractive index. This means that the refractive index causes changes in wavelength. This is necessary to be able to use the structure as a biosensor. The input end of a biosensor based on the structure designed here would be connected with nano fiber to provide a channel for incident light, and its output would be connected with JY Confocal Raman Microscopy, which would be used to detect an output signal [32]. The biosensor could be used for human health monitoring, such as glucose concentration detection [33], temperature monitoring [34,35,36], and hemoglobin detection [37,38]. Trace elements are also essential for the human body; the structure we designed could be used to detect the content of Na^+^ in our body. The relational expression between the concentration and refractive index is shown in [39]:

(5) $$n=1.3373+1.768\times 10^{-3}\frac{C*k}{393}-5.8\times 10^{-6}{\left(\frac{C\times k}{393}\right)}^{2}$$

Here, *C* indicates concentration in mgdL^−1^, and *k* = 50 is the concentration factor. Its results are shown in Figure 8b,c. With the concentration increase, refractive index of the structure changes from 1.36294 to 1.38587, and the curve has a redshift, which is identical to Figure 8a. When used for Na^+^ concentration monitoring, its sensitivity is 0.505 nm/mgdL^−1^.

**Figure 9:**
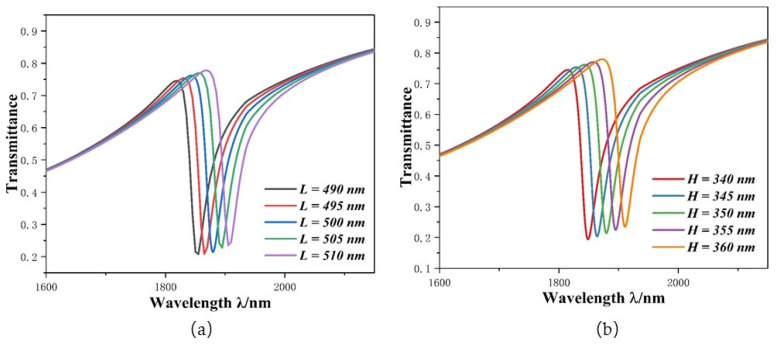
(**a**) Transmission spectra of *L*; (**b**) Transmission spectra of *H*.

Finally, during the fabrication of nanocomponents, errors of a few nanometers, due to poor accuracy or other reasons, can make the sensitivity less than the expected performance. Therefore, we conducted a five-nanometer error-simulation experiment for the parameters *L* and *H* for the greatest influence on sensitivity. Assuming the expected sensitivity of 2260 nm/RIU (the sensitivity was achieved under the parameters of refractive index experiment), in the experiment, *L* was changed from 490 nm to 510 nm at intervals of 5 nm and *H* was changed from 340 nm to 360 nm at intervals of 5 nm. The results are shown in Figure 9a,b. The transmission inclination shift to the right shows that the error has a high effect on sensitivity. It is calculated that every 1 nm error will cause a sensitivity change of about 24 nm/RIU. Therefore, the structure needs high precision control during production.

## 5. Conclusions

In this article, a nano-refractive index sensor structure is proposed that is composed of an MIM waveguide with a stub-1 and an orthogon ring resonator (ORR) with a stub-2. The Fano resonance of this structure is explained and the effect of structural parameters on transmission performance is studied. ORRS is a discrete state required to generate Fano resonance, and its *L* and *H* changes greatly affect the position of the dip (i.e., the sensitivity changes significantly). Stub-1 is a continuous state required to generate Fano resonance, and a change of parameter *h* causes a change of transmittance, which will affect the value of *FOM*. Other parameters do not have a significant impact on performance. In the data studied, the best parameters settings are: *L* = 500 nm, *H* = 350nm, *l* = 250 nm, *d* = 353 nm, *φ* = 60°, *g* = 10 nm, and *h* = 140 nm. At this point, its sensitivity reaches 2260 nm/RIU with a *FOM* of 211.42 and the transmittance is as low as 0.214. With this structure, the proposed sensor for the detection of Na^+^ could achieve a sensitivity of 0.505 nm/mgdL^−1^. As a result, the experimental results provide a certain point of reference for the application of a MIM-based sensor in human monitoring.

## Figures and Tables

**Table 1:** Comparison with data from other literature.

Reference	Structure	Sensitivity	Operating Wavelength Range
This paper	Orthogon ring resonance with short column	2260 nm/RIU	1500 nm < *λ* < 2000 nm
[22]/2017	A side-coupled split-ring resonator	1400 nm/RIU	800 nm < *λ* < 1400 nm
[21]/2018	Split ring resonator with rectangular short column	1200 nm/RIU	800 nm < *λ* < 1400 nm
[20]/2018	Split rectangular ring resonator with rectangular	1800 nm/RIU	800 nm < *λ* < 1400 nm
[29]/2019	Symmetric M-type resonator with baffle	780 nm/RIU	800 nm < *λ* < 1600 nm
[28]/2020	Bow-tie resonator	2300 nm/RIU	800 nm < *λ* < 2300 nm
[30]/2020	Ag-air grating	2000 nm/RIU	800 nm < *λ* < 2000 nm
[31]/2021	A split-ring resonance cavity and a double symmetric rectangular stub waveguide	1328.8 nm/RIU	800 nm < *λ* < 1500 nm

**Table 2:** Comparison with data from different structures.

Structure	Sensitivity	FOM	Operating Wavelength Range
ORRS	2260 nm/RIU	211.42	1500 nm < *λ* < 2000 nm
ORRS without Stub-2	2200 nm/RIU	219.51	1500 nm < *λ* < 2100 nm
Square Resonators without Stub-2	2100 nm/RIU	231.81	1500 nm < *λ* < 2000 nm
